# A Role for Primary Care Pharmacists in the Management of Inflammatory Bowel Disease? Lessons from Chronic Disease: A Systematic Review

**DOI:** 10.3390/pharmacy8040204

**Published:** 2020-11-02

**Authors:** Sharmila S. Prasad, Kerith Duncanson, Simon Keely, Nicholas J. Talley, Therése Kairuz, Gerald J. Holtmann, Ayesha Shah, Marjorie M. Walker

**Affiliations:** 1 Faculty of Health and Medicine, School of Biomedical Science and Pharmacy, University of Newcastle, Callaghan, NSW 2308, Australia; simon.keely@newcastle.edu.au (S.K.); therese.kairuz@newcastle.edu.au (T.K.); 2 Priority Research Centre, Digestive Health and Neurogastroenterology, University of Newcastle, New Lambton Heights, NSW 2305, Australia; kerith.duncanson@newcastle.edu.au (K.D.); nicholas.talley@newcastle.edu.au (N.J.T.); marjorie.walker@newcastle.edu.au (M.M.W.); 3 Faculty of Health and Medicine, School of Health Science, University of Newcastle, New Lambton Heights, NSW 2305, Australia; 4 Faculty of Health and Medicine, School of Medicine and Public Health, University of Newcastle, New Lambton Heights, NSW 2305, Australia; 5 Faculty of Medicine, University of Queensland, Brisbane, QLD 4001, Australia; g.holtmann@uq.edu.au (G.J.H.); ayesha.shah@uq.edu.au (A.S.)

**Keywords:** chronic disease, inflammatory bowel disease, diabetes, asthma, primary care, pharmacist, intervention

## Abstract

**Background and aims:** Chronic disease, particularly inflammatory bowel disease (IBD), requires a multifaceted approach to managing patients, but it is apparent that primary care pharmacists are being underutilized. To demonstrate the benefits of pharmacist interventions in primary care, a systematic review was conducted of interventions in asthma and type 2 diabetes where pharmacists have a defined role in chronic disease management. We also explored potential opportunities for primary care pharmacists to deliver tailored care to patients with inflammatory bowel disease. **Methods:** The search strategy retrieved original research articles from seven databases; eligible articles were assessed for inclusion. Quality appraisal was performed independently by two reviewers. **Results:** Thirty-seven included studies were grouped into four categories of interventions: education/counseling (43%), medication management (34%), monitoring/follow-up (17%), and screening/risk prevention (6%). Education plus counseling was reported as the main intervention delivered by pharmacists. Three measurable outcomes were identified: clinical, humanistic (e.g., quality of life), and economic. Clinical outcomes (63%) were reported more commonly than humanistic (26%) and economic (11%) outcomes. Pharmacist interventions led to statistically significant improvements in control of disease, severity, and medication adherence, as well as improvements in overall patient satisfaction, quality of life among patients with asthma and type 2 diabetes. **Conclusion:** As one of the most accessible sources of primary health care, pharmacists are well-placed to minimize the impact of chronic diseases on patients and communities. Evidence suggests there are opportunities for primary care pharmacists to play a more active role in the management of chronic diseases such as IBD.

## 1. Introduction

Chronic diseases, also referred to as non-communicable diseases (NCD), are the leading causes of mortality and disability globally [1]. The World Health Organization (WHO) defines a chronic disease as “diseases of long duration and generally slow progression” [2], which often means lifelong disease management, reduced quality of life, and poor mental health in these patients [1,2]. It is estimated that one in five people suffer from more than one chronic disease [3,4] and optimal management of the chronic disease is one of the most pressing challenges for healthcare systems.

Inflammatory bowel disease (IBD) is a group of chronic diseases including Crohn’s disease (CD) and ulcerative colitis (UC) [5]. IBD is generally diagnosed by early adulthood and is associated with chronic pain and progressively worsening disease. About 80% of patients require at least one surgical intervention in their lifetime (more commonly seen in Crohn’s disease) along with a series of adaptations to treatment regimens, fluctuating symptoms, and extra-intestinal manifestations that together cause severe detriment to the quality of life [6,7]. Multidisciplinary management of IBD is of proven value [8].

This high level of morbidity associated with chronic diseases has a substantial impact on individuals, their families and carers, communities, healthcare professionals, and the health system. Effective chronic disease management requires a holistic approach that is patient-oriented with the aim of reducing premature mortality and morbidity through multidisciplinary collaboration between primary, secondary, and tertiary sectors [1,9]. Two chronic diseases, type 2 diabetes and asthma, are known to be well managed in Australia, New Zealand, Canada, and the United Kingdom by primary care pharmacists working collaboratively within multidisciplinary healthcare systems [10,11].

Although all chronic diseases require a similar approach to management, namely, to improve patients’ quality of life, prevent complications, and reduce the burden of disease, IBD management is primarily dealt with by gastroenterologists [8]. Many of the key services are accessible only through secondary or tertiary care settings, even though there is evidence for a concerted multidisciplinary care approach in IBD management [12,13,14]. A recent systematic review highlighted the importance of pharmacists as primary care providers in the management of patients with chronic gastrointestinal diseases [8]. Their integration into the multidisciplinary primary care framework could potentially improve the management of IBD by actively participating in some of the essential preventative interventions.

To date, there are no published studies that evaluate the impact of pharmacist interventions in the primary care management of IBD. However, considerable research is available for type 2 diabetes and asthma, two chronic diseases that are predominantly managed in primary care through a collaborative multidisciplinary approach by General Practitioners (GPs) and pharmacists. Therefore, the primary aim of this review was to examine interventions provided by pharmacists in the management of type 2 diabetes and asthma and demonstrate the benefits of their interventions through measurable clinical, humanistic, and economic outcomes. Secondly, drawing on these examples, we discuss potential opportunities for pharmacists in primary care to deliver tailored care to patients with IBD as a means of addressing the current gap in the literature and contributing to the management of this chronic disease.

## 2. Materials and Methods

This systematic review follows the PRISMA (Preferred Reporting Items for Systematic Reviews) guidelines and is supported by the use of a PRISMA 2009 checklist (Table S1) [15].

### 2.1. Search Strategy and Study Eligibility

A systematic search of articles reporting original research in seven bibliographic databases and of the grey literature was conducted to identify studies relating to pharmacist interventions in type 2 diabetes and asthma, namely, International Pharmaceutical Abstracts, MEDLINE, SCOPUS, CINAHL, EMBASE, and PsycINFO; the search of these databases was conducted between September 2018 through to November 2019. An additional search in the Cochrane database was carried out to identify relevant systematic reviews.

Studies focusing only on secondary or tertiary care, review articles, non-original studies, notes, commentaries, and editorials were excluded. Inclusion criteria were articles published in English, which described the impact of pharmacist intervention in asthma or diabetes. No date restrictions were imposed. The ECHO model (economic, clinical, and humanistic outcomes) was used for the classification of interventions as this represents a conceptual framework for effective clinical practice improvements, logically compelling and more scientifically rigorous than a unidimensional approach to outcome studies [16]. Outcomes had to be measurable and occur in a primary care setting, as defined by the Australian Department of Health—National Primary Health Care Strategic Framework, namely, home- or community-based settings, such as community pharmacies, general practices, other private practices, and community health centers [4].

The search strategy was finalized in consultation with a senior research librarian using the following three Medical Subject Headings (MeSH): disease (diabetes OR asthma OR chronic disease), setting/profession (pharmaceutical care OR community pharmacy OR pharmacist*), and management (intervention* OR preventative health service* OR ((disease or medication) adj3 manag*) OR health education OR health promotion). See Table S2 for the full search strategy of Medline.

Manual searches of all published editorials, review articles, and reference lists from potentially relevant studies were conducted to identify additional studies. Grey literature was assessed and included websites of the Australian Journal of Pharmacy, Australian Pharmacist, and relevant professional/industry journals of countries published in other countries. Furthermore, a manual bibliographic search of conference abstracts arising from the systematic database search was performed. A word search of the internet was conducted using Google™ (word search: pharmacist intervention in asthma/diabetes) and the first 100 results were reviewed.

Duplicate articles were removed after the title and abstract screening, which was completed by two independent reviewers (SP and KD). These reviewers also screened full-text articles to assess their eligibility based on the inclusion and exclusion criteria. Any disagreement between these reviewers was resolved via consensus and did not require a third reviewer.

### 2.2. Quality Assessment

Due to the wide range of experimental study designs, a quality appraisal was performed using appropriate quality appraisal tools from the Joanna Briggs Institute at the University of South Australia, namely, the checklists for Randomised Controlled Trials [17], Quasi-Experimental studies (non-randomized experimental studies) [18], and Observational study designs using the Cohort studies checklist [19]. Responses to the appraisal questions (i.e., checklist) were allocated a 3-point ordinal scoring scale (2 = yes, 1 = partially, and 0 = no). The same two reviewers (SP and KD) independently evaluated the quality of each of the study criteria (Supplementary Material). Cohen Kappa [20] was used to indicate the degree of agreement between the two reviewers regarding the quality of this review. No studies were excluded due to poor quality (scores of less than 50%). Each eligible study included in this review was also assessed for the ‘risk of bias’ using the modified Cochrane Collaboration tool and graded (high risk, low risk, or some concerns) based on the combined grading of five domains (selection, performance, attrition, reporting, and other) [21].

### 2.3. Data Extraction

The study characteristics of the included articles were systematically recorded using a customized Excel^®^ spreadsheet and included bibliographic references (first author, year, and reference number), study design/methodology, interventions (monitoring/follow-up, medication management/review, screening/risk prevention, education plus counseling), outcomes measured (clinical/humanistic/economic), key findings (results and conclusion), level of evidence, and comments. The process of extracting data was undertaken by two reviewers (SP and KD). A meta-analysis was not feasible due to the heterogeneity of the studies.

## 3. Results

### 3.1. Study Selection

**Figure 1:**
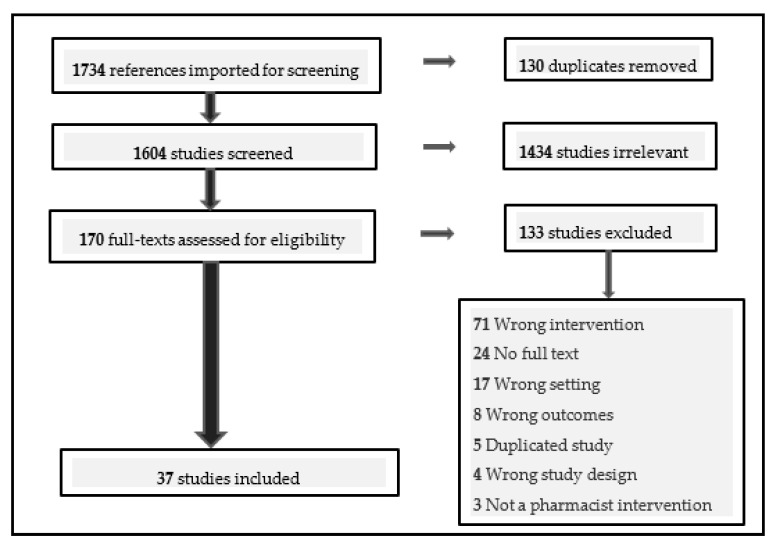
PRISMA (Preferred Reporting Items for Systematic Reviews) flow diagram that describes the process and results of the systematic search undertaken.

There were 1734 articles selected for screening, of which, 1604 remained after duplications had been removed. After initial abstract screening for relevance, 170 full-text studies were assessed for eligibility. After screening and quality appraisal, 37 studies were included in this review. See Figure 1 PRISMA flow chart and Table S3 for a summary of the characteristics of the included studies.

### 3.2. Assessment of Quality

Nineteen articles were deemed ‘high quality’ based on a quality score between 75% to 100%, of which seven were randomized controlled trials [22,23,24,25,26,27,28], 11 were quasi-experimental [29,30,31,32,33,34,35,36,37,38,39], and one was a cohort study [40]. Eighteen studies (with scores ranging between 50% and 74%) were of medium quality; of these, seven were randomized controlled trials [41,42,43,44,45,46,47], eight quasi-experimental designs [48,49,50,51,52,53,54,55], and three were cohort studies [56,57,58]. A Cohen kappa index score of 0.62 demonstrated a degree of substantial agreement between the two reviewers (SP and KD). All included studies had obtained ethical approvals.

### 3.3. Description of Studies

**Figure 2:**
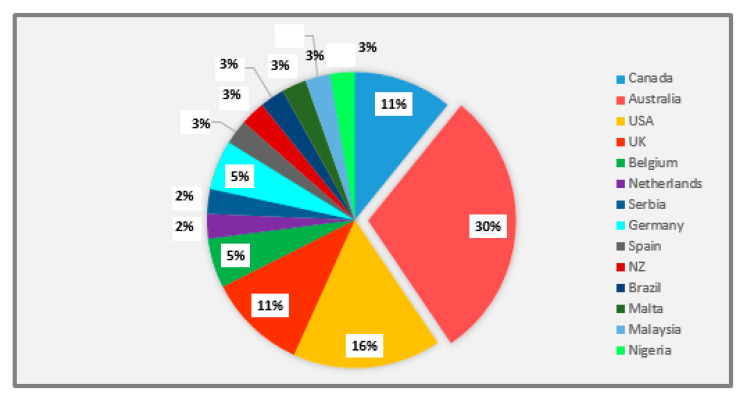
Geographical distribution of included studies on primary care management of Type 2 diabetes or asthma.

Most (87%; *n* = 32) of the included studies were of an experimental design including randomized controlled trials (38%; *n* = 14) [22,23,24,25,26,27,28,41,42,43,44,45,46,47], intervention studies (40%; *n* = 15) [30,31,33,34,35,36,37,38,39,48,49,50,51,52,55], quasi-experimental study (3%; *n* = 1) [32], before-and-after studies (3%; *n* = 1) [29], and time series study design (3%; *n* = 1) [53]. The remaining studies (13%; *n* = 5) were observational ones with cohort study design [40,54,56,57,58]. The distribution of studies for asthma and diabetes was almost the same with 19 (51%) studies related to asthma [23,27,30,32,34,38,39,42,43,45,46,47,49,50,51,52,54,57] and 18 (49%) studies to diabetes [22,24,26,28,29,31,32,33,35,36,37,40,41,48,53,55,56,58]. Studies originated from Australia (*n* = 11) [23,33,35,36,37,38,43,47,52,54], the United States of America (USA) (*n* = 6) [26,40,45,53,56,58], Canada (*n* = 4) [22,29,46,49], the UK (*n* = 4) [31,41,42,55], New Zealand (*n* = 1) [50], Belgium (*n* = 2) [27,28], Spain (*n* = 1) [44], Netherlands (*n* = 1) [57], Germany (*n* = 2) [39,51], Serbia (*n* = 1) [34], Malta (*n* = 1) [25], Nigeria (*n* = 1) [48], Brazil (*n* = 1) [32] and Malaysia (*n* = 1) [24] (Figure 2). Both rural and metropolitan settings were represented.

### 3.4. Types of Intervention by Pharmacists

**Figure 3:**
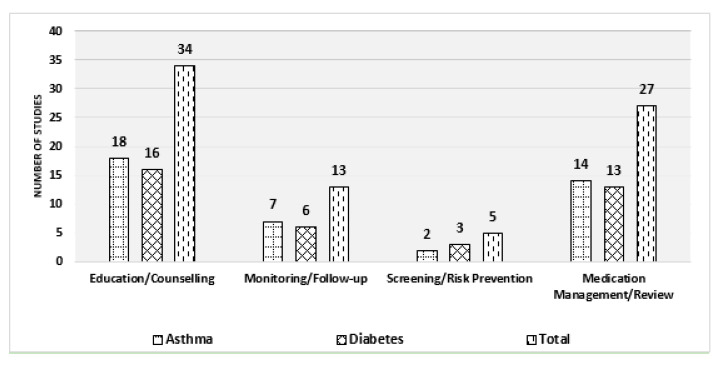
Comparison of interventions between asthma and diabetes.

As part of the inclusion criteria for this review, all interventions had to be performed by pharmacists within a primary care setting. Interventions included at least of one the following four key components: education plus counselling (43%; asthma *n* = 18, diabetes *n* = 16) [23,24,25,26,27,28,31,32,33,34,35,36,37,38,39,40,41,42,43,44,45,46,47,48,49,50,51,52,53,54,55,56,57,58], medication management/review (34%; asthma *n* = 14, diabetes *n* = 13) [22,23,26,27,28,29,30,32,33,34,35,36,37,38,40,44,45,46,47,50,52,53,55,56,57,58], monitoring/follow-up (17%; asthma *n* = 7, diabetes *n* = 6) [25,26,30,31,32,34,36,39,40,44,50,51,55], and screening/risk prevention (6%; asthma *n* = 2, diabetes *n* = 3) [29,30,36,40,45]. Most studies (78%; *n* = 29) applied multiple interventions, although only two studies implemented all four components [36,40]. The average number of interventions performed was two (20 studies) [22,23,25,27,28,31,33,35,36,37,38,39,46,47,51,52,53,56,57,58] with eight studies each performing one [24,29,41,42,43,48,49,54] or three interventions (Figure 3) [26,30,32,34,44,45,50,55].

**Figure 4:**
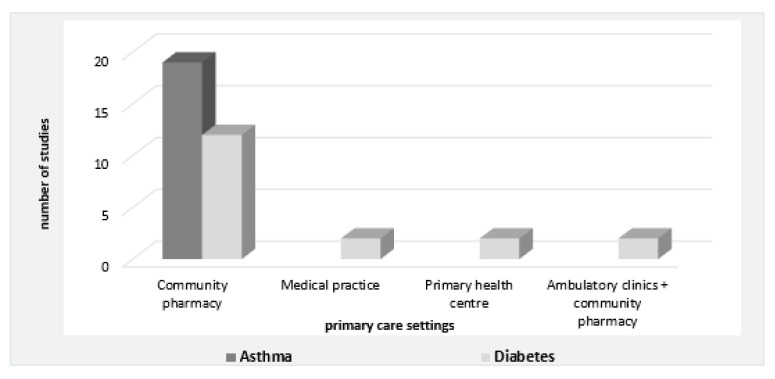
Classification of interventions into primary care settings.

The type and number of pharmacist interventions for asthma and diabetes were similar and education plus counseling was clearly the most common intervention for each of these chronic diseases. Other interventions were equally represented among both diseases. Approximately 85% (*n* = 31) of the interventions occurred in a community pharmacy and 15% were conducted in medical practices (*n* = 2), primary health centers (*n* = 2), and pharmacy/ambulatory clinics (*n* = 2). Although type 2 diabetes interventions were performed in all of the primary care settings mentioned, asthma interventions only occurred in community pharmacies (Figure 4). Furthermore, pharmacist interventions were only performed in populations with existing conditions of asthma or diabetes. There were no studies that evaluated healthy or ‘at risk’ populations. Although education plus counseling was seen to be the most common, it was not possible to determine which intervention delivered the greatest benefits.

### 3.5. Measurable Outcomes of Pharmacist Interventions

Type 2 diabetes and asthma showed a similar trend for all three measured outcomes (Table 1). Overall, twenty-one studies reported only one outcome (clinical [24,26,28,30,31,32,34,37,44,45,48,49,50,51,52,53,54,55,57,58] or humanistic) [42] followed by two outcomes (clinical + humanistic: *n* = 10 [22,23,27,29,35,38,39,40,41,43] and clinical + economic: *n* = 2) [33,56]; four studies reported all three outcomes [25,36,46,47]. 

Key outcomes from the included studies were clinical (63%), followed by humanistic (26%) and economic (11%). In studies among patients with type 2 diabetes, just under half (48%) of the studies reported only clinical outcomes, while 58% reported two outcomes (of which 71% were clinical + humanistic and 29% were clinical + economic) and a quarter (25%) reported all three outcomes. Clinical outcomes included glycosylated hemoglobin (HbA1c) of ≤7%, fasting blood glucose levels (BGL), cardiovascular (CV) risk factors, blood pressure, and body mass index (BMI). As demonstrated in the RxING study, 51% of participants achieved target HbA1c of ≤7% at the end of the study with 95% CI and *p*-value of <0.001, with a change in the fasting blood glucose (BGL) of 4.1 mmol/L (95% CI and *p* = 0.007) [29]. In comparison, studies among patients with asthma reported just over half (52%) of the only clinical outcomes, 42% had two measured outcomes (included clinical + humanistic), and three quarters (75%) of the studies measured all three of the outcomes (Table 1). Outcomes were related to inhaler technique, preventer/reliever usage and ratio, peak expiratory flow (PEF), and hospitalization. As observed in a cluster-randomized trial [44] that reported enhanced asthma control in the intervention group with 95% CI and *p* < 0.001 (OR = 3.06), improved mean Asthma Control Questionnaire (ACQ) scores (*p* < 0.001), increased number of controlled asthma patients by 30.1% (*p* < 0.001), improved medication adherence by 40.3% (*p* < 0.001), and better inhaler technique (56%; *p* < 0.001). No significant changes were observed in the control group [44]. Humanistic outcomes studies included patient satisfaction (33%) and quality of life (67%), while economic outcomes related to cost-effectiveness and cost savings for medication and hospitalization, and willingness to pay (WTP). For example, a randomized study demonstrated overall cost-effectiveness of AU$43 with reductions in the number of glycemic episodes in the intervention group compared to control (95 % CI 0.22, 0.52 (OR 0.34), *p* = 0.001; 95 % CI 0.34, 0.86 (OR 0.54), *p* = 0.009) [33]. 

## 4. Discussion

The scope for pharmacist interventions continues to evolve and has certainly increased in chronic disease management in recent decades [1]. The review identified education plus counseling, medication management/review, monitoring/follow-up, and screening/risk prevention as key interventions performed by pharmacists for type 2 diabetes and asthma in primary care settings and highlighted the benefits of these interventions through the resulting measurable outcomes. While type 2 diabetes and asthma are very different chronic diseases, varying in drug therapy, management, and patient needs, both nevertheless have a similar reliance on primary care providers. The findings of the review are consistent with other published literature [62,63,64,65,66]; however, our study is the first to hypothesize that pharmacist interventions could be applied to the management of IBD. In particular, we suggest that screening/risk prevention, monitoring/follow-up, education plus counseling, and medication management provided by appropriately trained pharmacists could contribute to effective long-term management of IBD in a primary care setting. The management of IBD occurs predominately in secondary or tertiary care [8], which is in contrast to the management of type 2 diabetes and asthma, where primary care pharmacists have extensive involvement [1]. However, our review identified that pharmacist interventions in type 2 diabetes and asthma showed similar trends in improved patient outcomes, i.e., better disease control, improved patient quality of life, and adherence to therapy. Therefore, it is reasonable to relate the interventional benefits to potential opportunities for primary care pharmacists to deliver tailored care to IBD patients.

Interventions have a key role in chronic disease management [1]. The results of the review highlight the value associated with patient monitoring, disease control, and patient adherence to therapy in type 2 diabetes and asthma [1,10,59,60]. Although screening/risk prevention and monitoring/follow-up interventions were reported least frequently among patients with type 2 diabetes and asthma, they play an important role in the early detection and management of chronic diseases. For example, effective interventions led to positive outcomes from complications affecting patients with type 2 diabetes [22,24,33,37,53]; and pharmacist interventions empowered patients to recognize worsening symptoms of their asthma and improved disease control and severity [23,27,42,44,54]. Literature suggests that IBD patients do not receive preventative services at the same frequency as general medical patients [61,62], thus creating a need for a proactive role from healthcare providers. Several studies have highlighted the importance of adequate immunization, screening of psychological health, skin cancer, and osteoporosis in IBD patients [61,62,63]. As patients often present with non-specific symptoms in primary care, pharmacist involvement could allow for early screening, management of flares, and complications associated with IBD [64]. Vaccinations are a good example of a preventative health service that could be delegated to appropriately trained and accredited pharmacists as part of an intervention strategy in primary care. Pharmacist vaccination services are available in approximately 13 countries: Argentina, Australia, Canada, Costa Rica, Denmark, Ireland, New Zealand, Philippines, Portugal, South Africa, Switzerland, the United Kingdom, and the United States of America [65]. 

Education plus counseling are fundamental to optimizing care in chronic disease sufferers [2,66]. Patient-centered care, patient participation, and shared decision-making are important in the active partnership that patients have in their care [67]. However, without relevant information that is tailored to individual needs, patients cannot make informed choices or participate in decision-making and/or self-management [67,68]. This review demonstrated that tailored patient education plus counseling resulted in improved disease severity and control, inhaler usage, and technique for patients with asthma. A better understanding of the need for medication led to overall patient satisfaction, improved quality of life, and better adherence to therapy, lifestyle changes, and self-care activities for patients with asthma and type 2 diabetes. Medications are the basis of treatment for IBD, although patients often have poor adherence to medication [69,70]. Unfortunately, non-adherence to medication in IBD occurs in up to 45% of patients and is associated with an increase in disease activity, relapse, loss of response to therapy, higher morbidity and mortality, poor quality of life, and increased health costs [69,70]. However, interventions to improve medication adherence have consistently demonstrated improved health care outcomes and a reduction in total health care costs [60]. Therefore, we propose that through established remunerated pharmacy medication management, appropriately trained pharmacists could provide education plus counseling to IBD patients about their medications. Multidisciplinary collaborations between primary healthcare professionals, such as GPs and pharmacists, and secondary/tertiary healthcare professionals, for instance, nurses and gastroenterologists, could facilitate the delivery of valuable education plus counseling to IBD patients in key areas such as disease flares, psychological health, nutritional support, medication management, and preventative health. Existing care models, such as chronic disease management plans, could be utilized to complement primary-secondary-tertiary care collaborations that target the complex health care needs of IBD patients. However, pharmacists may need further education to gain adequate knowledge of and clinical experience with this disease for this to be effective in practice [71]. 

Chronic abdominal pain is a very common and debilitating symptom for IBD sufferers, with up to 70% of patients experiencing pain due to exacerbation of the disease [72,73,74]. Pain management, therefore, is an important therapeutic target for IBD therapy, although it is associated with risks and limitations [74]. Evidence shows that about 30% of IBD patients are prescribed an opiate medication during the course of the disease [72,73]. Opiate use in IBD patients can cause narcotic bowel, mask disease flares and most commonly, long-term use can lead to opiate dependency [74]. The review demonstrated the effectiveness of pharmacist-led interventions that targeted medication management and improved disease severity and control and better compliance to therapy in patients with type 2 diabetes and asthma. We suggest that similar disease-specific interventions may provide an opportunity for primary care pharmacists to address complex medication needs in IBD patients; proactive and tailored interventions addressing medication management among IBD patients could improve medication adherence, optimize the use of over-the-counter medicines, and contribute to therapeutic monitoring of prescribed medications.

This review has some limitations. Studies differed in their design, outcomes, and measurements, and this heterogeneity reduced our ability to make precise assessments or conduct a meta-analysis. Articles published in languages other than English were excluded, which meant that potentially relevant studies from developed as well as developing nations were not included. This review focused on two specific chronic diseases (type 2 diabetes and asthma) and may have overestimated or underestimated the impact of pharmacist interventions on these diseases. Although the extent of involvement of pharmacists may vary according to the country in which they practice, primary care pharmacists are well-positioned to play an important role in holistic approaches to chronic disease management including IBD.

## 5. Conclusions

The available data suggest that primary care pharmacists can contribute to the services that are required to manage patients with chronic diseases, such as type 2 diabetes, asthma, and IBD. This requires innovative multidisciplinary models and collaboration between healthcare professionals in various health care settings. Considering the complex health care needs of IBD patients, primary care pharmacists are uniquely placed to complement existing care models and ultimately may help to improve patient outcomes. The introduction of pharmacist-delivered interventions requires integrated care models with defined accountabilities and communication/reporting pathways for all providers. It is evident that for pharmacist-delivered services to be valuable in the routine clinical setting careful integration and coordination are required.

## Figures and Tables

**Table 1:** Types of outcomes measured through pharmacist interventions.

Disease	Clinical	Humanistic	Economic	Clinical + Humanistic	Clinical + Economic	Clinical + Humanistic + Economic
Diabetes	10	0	0	5	2	1
Asthma	10	1	0	5	0	3
**Total**	**20**	**1**	**0**	**10**	**2**	**4**

## Supplementary Materials

The following are available online at https://www.mdpi.com/2226-4787/8/4/204/s1, Table S1: PRISMA 2009 Checklist, Table S2: Medline Search Strategy, Table S3: Summary of Included Studies.

## Disclaimer

S.K.: Grant/research support: Cancer Institute NSW (Career Development Fellowship), National Health and Medical Research Council (Project Grant), Commonwealth Diagnostics International (biomarkers for FGIDs), Syntrix Biosystems (contract research—drug delivery). Anatara Lifesciences (Advisory Board/Funded research). Gossamer Bio (Advisory Board/Funded research). N.J.T.: Grant/research support: Rome Foundation; Abbott Pharmaceuticals; Datapharm; Pfizer; Salix (irritable bowel syndrome); Prometheus Laboratories Inc (Irritable bowel syndrome [IBS] Diagnostic); Janssen (constipation). Consultant/Advisory Boards: Allakos (IBS), Adelphi Values (functional dyspepsia [patient-reported outcome measures]; [Budesonide]); GI therapies (chronic constipation [Rhythm IC]); Allergens PLC; Napo Pharmaceutical; Outpost Medicine; Samsung Bioepis; Yuhan (IBS); Synergy (IBS); Theravance (gastroparesis). Patent holder: Biomarkers of irritable bowel syndrome; Licensing Questionnaires (Mayo Clinic Talley Bowel Disease Questionnaire—Mayo Dysphagia Questionnaire); Nestec European Patent (Application No. 12735358.9); Singapore “Provisional” Patent (NTU Ref: TD/129/17 “Microbiota Modulation of BDNF Tissue Repair Pathway). M.M.W.: Grant/research support: Prometheus Laboratories Inc (Irritable bowel syndrome [IBS] Diagnostic), Commonwealth Diagnostics International (biomarkers for FGIDs).

## Abbreviations

IBD—Inflammatory bowel disease; QoL—Quality of life; HbA1c—Glycosylated hemoglobin; RCT—Randomized controlled trial; BGL—Blood glucose level; CV—Cardiovascular; BMI—Body mass index; PEF—Peak expiratory flow; WTP—Willingness to pay.
