# Supplementary material for: A Role for Primary Care Pharmacists in the Management of Inflammatory Bowel Disease? Lessons from Chronic Disease: A Systematic Review
Source: Pharmacy (Basel). 2020 Nov 2;8(4):204. doi: 10.3390/pharmacy8040204 (PMC7712000; doi:10.3390/pharmacy8040204)
Supplement: Supplementary file 1 [file pharmacy-08-00204-s001.zip › Characteristics of included studies_SuppInfo 1.docx]

**Supplementary Information 3**: Summary of the included studies

| **Reference (first author /year)** | **Study Design** | **Type of Intervention** | **Outcome Measured** | **Results** | **Key Findings** | **Risk of Bias/Level of evidence/Comments** |
| --- | --- | --- | --- | --- | --- | --- |
| **Asthma Studies** | | | | | | |
| Armour, C. et al. 2007 **^29^** | Randomised controlled trial | education/  counselling, medication management/ review | Clinical + Humanistic | Improved asthma control; patients receiving intervention improved from severe to not severe vs. control; improved adherence to preventer; decrease in reliever usage; improved QoL and knowledge in intervention group. | Pharmacist-delivered asthma care based national guidelines improves asthma control in patients. | Low risk (H – 81%): Patients were selected on poor control therefore whether positive results may affect generalisability of the results to a whole population; |
| Barbanel, D. et al. 2003 **^48^** | Randomised trial | education/ counselling | Humanistic | Baseline scores were similar in both groups; After 3 months, symptom scores significantly improved in the intervention group and marginally worsened in the control group. | Self-management programmes delivered by community pharmacists can improve asthma control in patients. | Some concern (M – 65%): Sample size is small; lack of blinding and outcome measures other than a validated health status scale; |
| Basheti, I. A. et al. 2008 **^49^** | Randomised parallel group | education/ counselling | Clinical + Humanistic | At baseline, patients in both groups showed poor inhaler techniques; at 6 months, techniques improved in intervention group; significant improvement in asthma severity as well. | Simple educational intervention delivered by community pharmacists is effective in asthma control and management. | Some concern (M – 71%): Randomisation and blinding provided assurance of accurate data collection. |
| Bereznicki, B. et al. 2013 **^36^** | Randomised intervention study | screening, monitoring, medication management/review | Clinical | Significant improvements in the preventer to reliever ratio after the intervention period (P < 0.0001) in each group. | Pharmacists can effectively identify patients with suboptimal asthma management that can be referred to GP for review. | Low risk (H – 100%): No data on precise pattern of use/adherence thus assumed to be dispensed equal to consumption. |
| Cordina, M. et al. 2001 **^31^** | Randomised controlled trial | education/  counselling, monitoring | Clinical + Humanistic + Economic | HRQoL of intervention group improved at 12 months; In the control group, PEF significantly decreased whereas inhaler technique improved in the intervention group. | Community programs by pharmacists in primary care has a positive impact on the vitality of patients with asthma, inhaler technique and PEF. | Low risk (H – 75%): Small sample size; lack of blinding; high rates of drop outs. |
| Diamond, S. A. et al. 2001 **^55^** | Intervention study | education/  counselling | Clinical | Significant decreases in the frequency of daytime asthma symptoms, nocturnal symptoms & short acting beta2-agonists usage. | Educational intervention can produce significant short term improvements self-management behaviour. | Some concern (M – 58%): Practical limitations - lack of individual placebo devices necessary for training each patient with asthma on the correct technique. |
| Emmerton, L. et al. 2003 **^56^** | Intervention study | monitoring, medication management/review, education/ counselling | Clinical | Clinical outcomes included reduced bronchodilator use, improved symptom control in 2/3 patients; asthma-specific QoL changes were more positive and correlated well with clinical indicators. | Community pharmacists with training and support, are highly capable of implementing a specialist asthma management service, with positive outcomes. | Some concern (M – 72%): Small sample size thus caution against generalisation |
| Garcia-Cardenas, V. et al. 2013 **^50^** | Randomised controlled trial | monitoring, medication management/review, education/ counselling | Clinical | Intervention resulted in enhanced asthma control; intervention group had improved medication adherence & inhaler technique. | Community pharmacists can increase controlled asthma patients compared to usual care. | Some concern (M – 73%): Small sample size - limited the power of the study to detect small. |
| Hammerlein, A. et al. 2011 **^57^** | Intervention study | monitoring, education/ counselling | Clinical | All patients benefited from pharmacists’ intervention regardless of their former training experiences. | Community pharmacy-based pharmacists can significantly supplement doctor-based education in inhalation technique. | Some concern (M – 61%): Selection bias cannot be fully excluded. |
| Klassing, H. M. et al. 2018 **^51^** | Randomised controlled trial | screening, medication management/review, education/ counselling | Clinical | Control group had significantly more influenza vaccinations; interventions showed a higher rate of influenza vaccination over the phone call; no significant improved found for pneumococcal. | Community pharmacists through effective intervention can increase immunisation rates in patients with specific needs. | Some concern (M – 69%): Large loss of participants due to incorrect contact details; self-reporting disease state as opposed to validating with GP. |
| Kovacevic, M. et al. 2018 **^40^** | Intervention study | monitoring, medication management/review, education/ counselling | Clinical | Better asthma control was significantly associated with higher adherence level, lower concerns on medication use, & knowledge of triggers; better asthma control was achieved in 60% of patients. | Community pharmacist education improved knowledge of asthma and medications, beliefs/attitudes towards asthma medications. | Low risk (H – 78%): No control group; participants health literacy not assessed. |
| Kritikos, V. et al. 2007 **^44^** | Intervention study | medication management/review, education/  counselling | Clinical + Humanistic | Significant decrease in the proportion of patients with severe asthma/poor control, improvement in inhaler technique & asthma knowledge for both A & B compared to C. | Education intervention by pharmacists appears to be more effective than usual care in improving clinical and humanistic asthma outcomes. | Low risk (H – 100%): Sampling bias, selection bias, small sample size, short follow-up affecting generalisability. |
| Lemay, K. S. et al. 2015 **^58^** | Intervention study | medication management/review, education/  counselling | Clinical | Pharmacists provided 22,909 clinical pharmacy interventions over the service; patients had a high and ongoing need for interventions. | Pharmacists were able to use clinical judgement to assess patients and provide clinical pharmacy interventions. | Some concern (M – 53%): Self-reporting of interventions by pharmacists; interpreted differently despite the simplistic checklist design. |
| McLean, W. et al. 2003 **^52^** | Randomised controlled trial | medication management/review, education/counselling | Clinical + Humanistic + Economic | Overall quality of life, symptoms, emotional function improved; initial knowledge scores doubled; emergency room & medical visits decreased. | Pharmacists produced improvements in clinical, economic & humanistic outcome. | Some concern (M – 56%): Level of effect of UC was possibly already at elevated level thus caution with generalisability. |
| Mehuys, E. et al. 2008 **^33^** | Randomised controlled trial | medication management/review, education/  counselling | Clinical + Humanistic | Intervention had significantly increased the ACT score after 6 months compared with usual care. | Pharmacists can significantly improve therapeutic outcomes in adult asthma patients. | Low risk (H – 83%): Straightforward inclusion criteria pleads for generalisability of the results. |
| Ottenbros, S. et al. 2014 **^63^** | Cohort study | medication management/review, education/  counselling | Clinical | All problems decreased additionally to the CG within the total asthma or COPD population from the IG; decreased obsolete, contraindicated medication & lower use of powder inhalers. | Community pharmacists actively providing comprehensive pharmacy care could improve effective treatment in asthma. | Some concern (M – 68%): No disposition of clinical outcomes; IG pharmacists not randomly selected. |
| Saini, B. et al. 2004 **^53^** | Randomised controlled trial | medication management/review, education/  counselling | Clinical + Humanistic + Economic | Significant reduction in asthma severity in the intervention group; statistically significant improvement in perceived control of asthma & asthma-related knowledge. | Specialized asthma care model offers community pharmacists an opportunity to contribute toward improving asthma management. | Some concern (M – 69%): Does not meet standard of a rigorous RCT; sample size is small thus results may not be representative of the population of asthmatics. |
| Saini, B. et al. 2011 **^60^** | Randomised controlled trial | education/  counselling | Clinical | Asthma knowledge significantly improved as a result of the service; improvement retained for at least 12 months after the service. | Improvements in knowledge, achievable & sustainable with targeted educational pharmacist interventions. | Some concern (M – 61%): Difficult to relate knowledge any particular intervention thus no direct measurement. |
| Schulz, M. et al. 2001 **^45^** | Intervention study | monitoring, education/  counselling | Clinical + Humanistic | Pharmaceutical care led to significantly improved inhalation technique; asthma-specific quality of life and the mental health summary score also improved in intervention group. | Pharmacist interventions have laid a basis for appropriate drug use, health attitudes, and health behaviour that improves the self-management. | Low risk (H – 100%); Control pharmacists may have engaged in counselling at 6mth due to tendencies for improvement in this group. |
| **Diabetes Studies** | | | | | | |
| Al Hamarneh, Y. N. et al. 2013 **^35^** | Pragmatic before-after trial | medication management | Clinical + Humanistic | HbA1c reduced at baseline; fasting BGL reduced; 51% of patients achieved target HbA1c of <7%. | Prescribing pharmacists showed marked clinical improvements in glycaemic control similar to physician-led studies. | Low risk (H- 78%): 26-week follow-up: relatively short period; response rate for QoL questionnaires were low. |
| Al Hamarneh, Y. N. et al. 2017 **^28^** | Randomised controlled trial | risk prevention, monitoring, medication management | Clinical + Humanistic | Estimated CV risk reduced by 0.4% in control group & 5.7% in intervention group in 3 months; | Showed significant improvement in CV risk & Tx regimens through pharmacist intervention. | Low risk (H – 85%): 3-month follow-up is relatively short, possible to imply the effects of the intervention could be short lived; Blinding was not possible due to the nature of the study. |
| Ali, M. et al. 2012 **^47^** | Randomised controlled trial | education/ counselling | Clinical + Humanistic | HbA1c reduced in intervention group; BP fell in intervention group with no significant difference in control group; similar changes in BMI & BGL for intervention group. | Community pharmacist involvement can yield improvements in type 2 diabetes management seen by improved primary & secondary outcomes of the study. | Low risk (M – 73%): Partial target recruitment despite 1 year of recruitment - may be due to specific inclusion criteria; |
| Bello, S. et al. 2012 **^54^** | Randomised intervention study | Education/ counselling | Clinical | Reduction in BMI, HbA1c and FBS. | Direct involvement of pharmacists in the care of diabetic patients in such primary care setting can significantly improve the QoL and reduce mortality caused by the disease. | Some concern (M – 58%): Sample size is small; lack of blinding and outcome measures. |
| Benedict, A. W, et al. 2018 **^46^** | Cohort study | risk prevention, monitoring, medication management, education/  counselling | Clinical + Humanistic | No significant difference in baseline for both groups; HbA1c values were significantly improved in intervention group vs. control at 3 months and 6 month intervals. | Clinical pharmacist can improve drug therapy management in type 2 diabetes patients; allow for access to services in primary care. | Low risk (H – 93%): HbA1c results not available for every patient, data from last observation used thus, may have been too generalised. |
| Bliss, E. et al. 2001 **^37^** | Intervention study | education/  counselling, monitoring | Clinical | Showed improvement in control. Overall the service was well accepted and was of value to both patients and health care providers. | Regular HbA1c testing combined with education helped poorly controlled patients to gain better management of their diabetes. | Low risk (H – 78%): Sample size is small thus is limited in its generalisability. |
| Chow, E. P. et al. 2015 **^30^** | Randomised controlled trial | education/  counselling | Clinical | Significant increase in knowledge of T2DM and medication adherence in home-based intervention group; significantly lower HbA1c levels as well. | Pharmacist-led home-based interventions can significantly increase disease-related knowledge and medication adherence in diabetic patients. | Low risk (H – 75%): Sample size is small thus caution against generalisation. |
| Correr, C. J. et al. 2011 **^38^** | Non-randomised control study | medication management/review, monitoring, education/  counselling | Clinical | Intervention group had greater HbA1c reduction, fasting BGL even with adjusted baseline values; no significant difference between the groups for clinical measures. | Pharmacists can assess medication clinical outcomes and identify negative clinical outcomes; improving glycaemic control through optimising medication profile. | Low risk (H – 100%): non-randomisation is a key limitation - difficult to measure non-blinded sample; |
| Cranor, C. W. et al. 2003 **^62^** | Cohort study | medication management, education/  counselling | Clinical + Economic | Changes in glycosylated haemoglobin (A1c) and serum lipid concentrations, diabetes-related and total medical utilization and costs over time. | Patients with diabetes who received ongoing PCS maintained improvement in A1c over time, a decline in mean total direct medical costs. | Some concern (M – 73%): Missing and/or unreported clinical data, resulting in diminished cohort sizes over time. |
| Doucette, W. R. et al. 2009 **^32^** | Randomised controlled trial | monitoring, medication management/review, education/ counselling | Clinical | Patients receiving interventions significantly increased their engagement in a set of diet and diabetes sell-care activities. | Educational intervention in the community pharmacy can significantly improve self-management behaviour. | Low risk (H – 79)%: Small sample size - limited the power of the study to detect small. |
| Hendrie, D. et al. 2014 **^39^** | Intervention study | medication management/review, education/ counselling | Clinical + Economic | Significantly greater reductions in number of hyperglycaemic and hypoglycaemic episodes occurred in the intervention and was cost-effective. | Pharmacists’ management of care of patients with chronic diseases such as type 2 diabetes has the potential to improve patient care outcomes. | Low risk (H – 97%): Small sample thus study should be viewed as a pilot; attribution rate was substantial. |
| Krass, I. et al. 2007 **^41^** | Intervention study | medication management/review, education/ counselling | Clinical + Humanistic | Greater improvements in glycaemic control in intervention group; improvements in BP control and QoL. | Community pharmacists can contribute significantly to improving care and health outcomes in type 2 diabetes. | Low risk (H – 100%): Data retrieval was difficult for some patients from their GPs, though missing data was similar for both groups. |
| Krass, I. et al. 2006 **^42^** | Intervention study | medication management/review/ monitoring, education/ counselling, | Clinical + Humanistic + Economic | Continuity-of-care model demonstrated greater improvement in glycaemic control, negative attitude & understanding of diabetes for both groups. | Continuity-of-care model had a significant impact on clinical and humanistic outcome within community and clinic settings. | Low risk (H – 100%): Lack of power due to under recruitment of participants. |
| Krass, I. et al. 2005 **^43^** | Intervention study | medication management/review, education/  counselling | Clinical | Significantly improved self-reported non-adherence; overall prevalence of changes to the regimen was higher in the intervention group. | Community pharmacists trained in medication review in collaboration with providers improved adherence, reduced problems relating to accessing medications. | Low risk (H – 100%): Non-randomisation is a key limitation - difficult to measure non-blinded sample. |
| Mehuys, E. et al. 2011 **^34^** | Randomised controlled trial | medication management/review, education/  counselling | Clinical | Intervention significantly reduced HbA1c; largest impact observed when pharmacotherapy changes initiated by the physician were sustained with pharmaceutical care. | Beneficial effect of community pharmacist intervention in the clinical management of type 2 diabetic patients. | Low risk (H – 81%): Underestimation of the effect of intervention; hawthorne effect - control group can improve performance by virtue of participation. |
| Nkansah, N. T. et al. 2008 **^59^** | Retrospective time series study | medication management/review, education/  counselling | Clinical | Significant reduction in HbA1c observed; no change noted in weight/number of patients at goal blood pressure. | Integrating pharmacist into private physician practice significantly improved patient glycaemic control. | Some concern (M – 56%): Small sample size, no randomisation of participants. |
| Oyetayo, O. O. et al. 2011 **^64^** | Cohort study | medication management/review, education/  counselling | Clinical | A1C was not reduced significantly from baseline to 12 months; statistically significant reductions seen for fasting plasma glucose, triglycerides, and diastolic blood pressure; | Interventions performed by community pharmacists are effective in improving clinical outcomes in a Hispanic cohort with diabetes. | Some concern (M – 55%): Lack of pre-specified control group; high drop-out rate. |
| Wermeille, J. et al. 2004 **^61^** | Intervention study | monitoring, medication management/review, education/  counselling | Clinical | Reduction in HbA1c, BP and total cholesterol observed: patient knowledge was poor for oral hypoglycaemic therapy but improved. | Pharmacists are effective & well received by GPs and patients to deliver pharmaceutical care model. | Some concern (M – 56%): Small sample size, no randomisation of participants. |

**H – high quality; M – medium quality; ACT; asthma control test; COPD – chronic obstructive pulmonary disease; HRQoL – health related quality of life; CG – control group; IG – intervention group; CV – cardiovascular; BGL – blood glucose level, HbAlc – glycosylated haemoglobin; QoL – quality of life; BP – blood pressure; BMI – body mass index; FBS – fasting blood sugar.*
