# Supplementary material for: A Role for Primary Care Pharmacists in the Management of Inflammatory Bowel Disease? Lessons from Chronic Disease: A Systematic Review
Source: Pharmacy (Basel). 2020 Nov 2;8(4):204. doi: 10.3390/pharmacy8040204 (PMC7712000; doi:10.3390/pharmacy8040204)
Supplement: Supplementary file 1 [file pharmacy-08-00204-s001.zip › Medline search strategy_Supplementary information.docx]

**Supplementary Information 2**: Medline search strategy

| **#** | **Searches** | **Results** |
| --- | --- | --- |
| 1 | DIABETES MELLITUS/ or Diabetes Complications/ or diabetes.mp. or Blood Glucose/ | 618092 |
| 2 | asthma.mp. or ASTHMA/ | 160261 |
| 3 | chronic disease.mp. or Chronic Disease/ | 269180 |
| 4 | 1 or 2 or 3 | 1022199 |
| 5 | 1 or 2 | 774018 |
| 6 | pharmaceutical care.mp. or Pharmaceutical Services/ | 9132 |
| 7 | community pharmacy.mp. or Pharmacies/ | 11650 |
| 8 | Community Pharmacy Services/ or Pharmacists/ or pharmacist*.mp. | 32303 |
| 9 | 6 or 7 or 8 | 41131 |
| 10 | EARLY MEDICAL INTERVENTION/ or intervention*.mp. | 851912 |
| 11 | Health Services/ or preventative health service*.mp. | 23796 |
| 12 | ((disease or medication) adj3 manag*).mp. or Disease Management/ | 66467 |
| 13 | health education.mp. or Health Education/ or Patient Education as Topic/ | 156930 |
| 14 | health promotion.mp. or Health Promotion/ | 80704 |
| 15 | 10 or 11 or 12 or 13 or 14 | 1107333 |
| 16 | 4 and 9 and 15 | 1479 |
